# Swimming and Aquatic Activities: State of the Art

**DOI:** 10.2478/v10078-012-0018-4

**Published:** 2012-05-30

**Authors:** Yolanda Escalante, Jose M. Saavedra

**Affiliations:** 1 Facultad de Ciencias del Deporte. AFIDES Research Group. Universidad de Extremadura, Spain.

The concepts of “swimming” and “water activities” have evolved greatly since the first written document on swimming (Nikolaus Wynmann, 1538, “*Colymbetes, sive de arte natandi dialogus et festivus et iucundus lectu*” [“*The Swimmer, or A Dialogue on the Art of Swimming and Joyful and Pleasant to Read*”]). At its inception, swimming was understood simply as moving in water – first, in order to survive, and then, to compete. Today, the term clearly connotes swimming sports, and as such can be defined as an activity in which a person practices a regulated Olympic sport in order to move as fast as possible through the water thanks to the propulsive forces generated by arm, leg, and body movements overcoming the resistance the water presents to progress (Saavedra et al., 2003). The skills of swimming have been adapted to different contexts and participants. The result is that there are now a great diversity of additional water activities: aquatic Olympic sports (water polo, diving, and synchronized swimming), non-Olympic sports (lifesaving, fin swim,…), leisure activities (aqua-aerobic, fit swim,…), health activities (hydrotherapy, balneotherapy,…). The term “Aquatic Activities” covers all these plus swimming, and can be defined as motor activities performed in water for purposes that may be utilitarian, competitive, educational, therapeutic, or recreational.

With regard to research, swimming is probably the most studied of all sports (Barbosa et al., 2010). The first research of which we know was published in 1905 by Du Bois-Reymond. He used an elementary dynamometer to measure the passive hydrodynamic drag of a swimmer pulled through the water by a boat. The father of swimming research, however, is considered to be Thomas K. Cureton of the University of Illinois. Beginning in the 1930s, he published numerous scientific papers related to swimming (Colwin, 1993). The scientific consolidation of swimming studies was reflected in specific conferences of worldwide significance, such as the series of FINA World Sports Medicine Congresses, the first of which was held in 1969 (London, UK), and whose next edition (XVII) will be in December this year in Istanbul (Turkey). Another outstanding series of Conferences are those of the International Society of Biomechanics in Sport in which numerous works related to swimming are presented, with “Applied Sessions” specifically devoted to this sport. For instance, in this year’s (30th) edition to be held in Melbourne there will be two such Applied Sessions, one devoted to the influence of wave drag in swimming, and the other to applications of technology in swimming. The most important scientific congress in the swimming world is, however, the quadrennial International Symposium on Biomechanics and Medicine in Swimming whose first edition was held in 1970. In the latest (11th) edition held in Oslo (2010), more than 250 works were presented representing more than 25 countries (Kjendlie et al., 2010), and there has been a steady growth in the number relating to aquatic activities (in particular, there were 50 papers presented in this area in the Oslo meeting). The four scientific areas with most works presented were, in this order: Biomechanics, Physiology, Evaluation, and Training (Vilas-Boas, 2010). Many of the papers presented in the various conferences have subsequently been published in Journal Citation Report indexed journals. One observes in Table 1 that Swimming predominates over the other thematic research areas, and that, in recent years, research in aquatic activities other than swimming has increased considerably.

The research being conducted in the field of aquatic activities in general, and swimming in particular, has led the Journal of Human Kinetics to publish this special issue. It comprises 21 high quality articles involving researchers and academics from 11 countries (Belgium, Brazil, Canada, Croatia, Greece, Poland, Portugal, Serbia, Slovenia, Spain, and the United States of America). Each article has been rigorously peer reviewed by at least two referees from 11 countries in addition to the nine above (Australia, Bosnia and Herzegovina, Estonia, France, Germany, Greece, Hungary, Japan, Lithuania, Romania, Switzerland, the United Kingdom), involving over 40 researchers and 25 institutions. The articles are divided into two main blocks – competitive swimming (12 papers), and other aquatic activities (9 papers) corresponding to the following themes: aquatic exercise (3 papers), swim education (2 papers), synchronized swimming (2 papers), water polo (1 paper), and diving (1 paper). By scientific area, the papers may be categorized as: Biomechanics (7), Anthropometric (2), Education (2), Evaluation (2), Physiology (2), Training (2), Aquatic exercise (1), Psychology (1), Sociology (1) and others (1).

Finally, we wish to thank the Editor-in-Chief, Professor Dr Adam Zając, and the Technical and Statistical Editor, Professor Dr Adam Maszczyk for the trust and responsibility deposited in us for the preparation of this Special Issue. We hope and trust that it will meet with the approval of the readers of the Journal of Human Kinetics.

## Figures and Tables

**Table 1:** *Search results (18th April 2012) for different terms in the Web of Knowledge for the “Sport Sciences” and other categories*.

Term	n			Percentage (%)		

	Sport Sciences	Other	Total	Sport Sciences	Other	Total
Aqua-aerobics	1	0	1	0.02	0.01	0.01
Aquatic exercise	58	64	122	1.17	0.67	0.84
Diving	1	23	24	0.02	0.24	0.17
Fin swim	45	34	79	0.90	0.36	0.55
Hydrotherapy	29	545	574	0.58	5.74	3.97
Other term	23	21	44	0.46	0.22	0.30
Balneotherapy	5	246	251	0.10	2.59	1.73
Swimming	4779	8423	13202	96.00	88.69	91.21
Synchronized swimming	3	41	44	0.06	0.43	0.30
Water polo	34	100	134	0.68	1.05	0.93

					100.0	100.0
Total	4978	9497	14 475	100.00	0	0
